# Intrathecal administration of clonidine or yohimbine decreases the nociceptive behavior caused by formalin injection in the marsh terrapin (*Pelomedusa subrufa*)

**DOI:** 10.1002/brb3.287

**Published:** 2014-10-02

**Authors:** Christopher M Makau, Philemon K Towett, Klas S P Abelson, Titus I Kanui

**Affiliations:** 1 Department of Veterinary Anatomy and Physiology, University of NairobiP.O Box 30197-00100, Nairobi, Kenya; 2 Department of Experimental Medicine, University of Copenhagen, Faculty of Health SciencesBlegdamsvej 3B, Copenhagen N, DK-2200, Denmark; 3 South Eastern Kenya University, School of Agriculture and Veterinary SciencesP.O Box 170-90200, Kitui, Kenya

**Keywords:** Antinociception, nociception, testudines, *α*_2_-adrenergic receptors

## Abstract

**Background:**

The role of noradrenergic system in the control of nociception is documented in some vertebrate animals. However, there are no data showing the role of this system on nociception in the marsh terrapins.

**Methodology:**

In this study, the antinociceptive action of intrathecal administration of the *α*_2_-adrenoreceptor agonist clonidine and *α*_2_-adrenoreceptor antagonist yohimbine was evaluated in the African marsh terrapin using the formalin test. The interaction of clonidine and yohimbine was also evaluated.

**Results:**

Intrathecal administration of clonidine (37.5 or 65 *μ*g/kg) caused a significant reduction in the mean time spent in pain-related behavior. Yohimbine, at a dose of 25 *μ*g/kg, significantly blocked the effect of clonidine (65 *μ*g/kg). However, administration of yohimbine (40 or 53 *μ*g/kg) caused a significant reduction in the mean time spent in pain-related behavior. Intrathecal administration of yohimbine (53 *μ*g/kg) followed immediately by intrathecal injection of the serotonergic methysergide maleate (20 *μ*g/kg) resulted in a significant reversal of the antinociceptive effect of yohimbine.

**Conclusion:**

The present study documented the intrathecal administration of drugs in the marsh terrapin, a technique that can be applied in future studies on these animals. The data also suggest the involvement of both *α*_2_-adrenoreceptors and 5HT receptors in the modulation of nociception in testudines.

## Introduction

There are multiple pain-modulating systems in the central nervous system of most vertebrate animals. These include the opioidergic, noradrenergic, and serotonergic systems. The role of opioidergic systems in pain modulation has been investigated in many animal species. Several studies on the antinociceptive potential of opioid and opioid-like drugs in reptiles and in particular the red-eared slider turtles have been performed (Sladky et al. 2007, 2009; Baker et al. 2011; Mans et al. 2011). The studies reported effective antinociception of some opioids, particularly the mu-selective agents. The bulbospinal descending noradrenergic and serotonergic pathways, however, have to our knowledge not been investigated in the testudines. Both systems play important roles in the modulation of nociceptive information in primary afferent neurons in the spinal cord dorsal horn (Reddy and Yaksh 1980; Yaksh et al. 1981; Proudfit 1988). The use of *α*_2_-adrenergic agonists as adjuncts in pain management is promising because of their action at the central and peripheral sites (Kamibayashi and Maze 2000). The analgesic effects of *α*_2_-adrenergic agonists have been widely documented in both humans and animals (Boyd et al. 2001; Mader 2006; Mosley 2006; Giovannoni et al. 2009). In reptiles, *α*_2_-adrenergic agonists have been mainly used in combination with other analgesic agents and have been found to produce potent analgesia (Holz and Holz 1994; Heard 2001; Dennis and Heard 2002; Mader 2006 and Mosley 2006). Some of the commonly used *α*_2_-adrenergic agonists in reptiles are xylazine, medetomidine, and dexmedetomidine (Mosley 2006). Clonidine, a *α*_2_-adrenoceptor agonist, has been used in various clinical settings and has been shown to exert excellent analgesic effects, especially when administered epidurally or intrathecally (Förster and Rosenberg 2004; Strebel et al. 2004). Eisenach et al. (1998) reported that intrathecal, but not intravenous clonidine reduced experimental thermal or capsaicin-induced pain and hyperalgesia in normal volunteers indicating that the route of drug administration influences its efficacy. Clonidine, however, has a number of other pharmacological properties due to effects in the CNS (Sierralta et al. 1996). A distinct advantage of using alpha-2 agonists is that they are reversible, thus facilitating more rapid recoveries (Mosley 2006).

Although yohimbine is a classical *α*_2_-adrenergic receptor antagonist, studies have shown that it decreases formalin-induced pain in rats (Kanui et al. 1993; Shannon and Lutz 2000). Yohimbine has also been shown to possess agonistic action on serotonergic 5HT_1A_ receptors (Owen and Whitton 2003). The purpose of this work was to evaluate the antinociceptive/antagonistic actions of clonidine and yohimbine administered intrathecally in the marsh terrapin (*Pelomedusa subrufa*) using the formalin test and also to examine the interactions between the two drugs. It was hypothesized that clonidine would reduce the formalin-induced nociceptive behavior, and that the reduction would be reversed by yohimbine. It was further hypothesized that yohimbine in higher doses would reduce formalin-induced nociceptive behavior, and that the reduction would be reversed by the serotonin receptor antagonist methysergide maleate.

## Materials and Methods

### Animals

Fifty six marsh terrapins sourced from dry and hot districts of Eastern Kenya were used for validation, nociceptive, and antinociceptive experiments. Body weight, sex, age, and plastron length of each animal was recorded on arrival. The animals used were adult males and females with a mean body weight of 450 g and a standard deviation of 10 g. They were housed in a well-ventilated room, with translucent windows. The animals were kept in metallic tanks measuring 1.25 × 1.0 × 0.6 mol/L. The tanks were half-filled with sand and a centrally located plastic basin filled with water was provided. The basin took a larger area as the terrapins prefer living in water. The marsh terrapins were fed twice in a week on sliced raw meat and floating food sticks or leafy vegetables. Animals were housed under standard laboratory conditions with a 12/12 h light/dark cycle and at a temperature of 26–30°C. The animals were habituated to the laboratory conditions for 1 month before the start of the experiments. During this period, they were adapted to a previously described restraining procedure (Wambugu et al. 2010). The experimental animals were acquired and cared for in accordance with the guidelines published in the National Institutes of Health Guide and use of laboratory Animals (National institute of Health Publication No. 85–23 revised 1995). All animal protocols were approved by the Animal Care and Use Committee of our institution.

### Drugs

The drugs used were clonidine hydrochloride (*α*_2_-agonist), yohimbine hydrochloride (*α*_2_-antagonist/5HT agonist) and methysergide maleate (5HT antagonist) (all from Sigma). The aim of using methysergide maleate was to determine any interaction of 5-HT system with *α*_2_-antagonist (yohimbine). Clonidine hydrochloride and methysergide maleate were dissolved in 0.9% saline. Yohimbine was dissolved in dimethyl sulphoxide (DMSO). Drugs or vehicles were administered intrathecally in a volume of 100 *μ*L using a 30-gauge needle. The dosages used were based on preliminary investigations from our laboratory. Fresh preparations of drugs were always used.

### Intrathecal administration

Lidocaine hydrochloride (100 *μ*L) was used to develop intrathecal technique of drug injection in the marsh terrapin. Six marsh terrapins were used for developing this technique. A metal rod was used to gently rub the animal at its back to make it relax. The head was then held at the neck region and an injection was made by gently inserting a 30-gauge, 1-cm long needle at the termination of the occipital process in line with the midline of the head. The needle was inserted, at an insertion angle of about 45° until atlanto-occipital membrane was hit. The needle was pushed almost half way its length to pass through the atlanto-occipital membrane into subarachnoid space where the drug was delivered. Prior to drug injection aspiration was done in order to avoid intravascular injection. Immediately the needle went through the atlanto-occipital membrane, the animal normally reacted by jerking its head. The general behavior and muscle tension was observed and recorded.

Prior to drug injections one animal was used to validate the intrathecal injection technique. The animal was injected intrathecally with Evans blue dye and then killed by injecting sodium pentobarbitone (200 mg/kg) intravenously into the jugular vein. The corneal reflex (blinking response) was performed to ascertain whether the animal was dead or alive and its absence indicated that it was dead (Torpyt et al. 2008). Once the animal was confirmed dead, it was placed on a dissecting dish and slit with the use of a scalpel at the point where Evans blue was injected. Pictures of the sections were taken immediately and the location of the dye identified.

### Formalin test

The animal was restrained on a stand with a string tied round its shell and then positioned facing away from the investigator, that is, facing the wall (Wambugu et al. 2010). This form of restraint seemed comfortable because the animal appeared relaxed. The procedure was performed in a sound attenuated room. The animal was then injected with 100 *μ*L of 8% formalin into the inter-claw space of the hind limb using a micro-liter syringe and a 29-gauge needle. The controls for formalin were given 100 *μ*L of saline (0.9% NaCl) or dimethylsulphoxide (DMSO) in a similar manner to that of formalin. The total time spent lifting the injected limb (referred to as hind limb withdrawal) was recorded. Recording was performed in 12 blocks of 5 min and the data were recorded as total time spent in pain-related behavior after the injection of formalin or vehicle. Animal reuse was allowed only after at least 2 weeks wash-out period, and in that case injection was performed on an alternative paw. The experiments were always performed at a room temperature of 26–28°C, and between 10.00 a.m. and 2.00 p.m.

### Antinociceptive testing

The animals were randomly grouped in eight groups each consisting of six animals. Each group was assigned a certain dosage level of the drug being tested. The injection site was aseptically prepared before the drugs were injected intrathecally. Six animals per dose were injected with clonidine (18.75, 37.5 or 65 *μ*g/kg), yohimbine (25, 40 or 53 *μ*g/kg), a combination of clonidine (65 *μ*g/kg) and yohimbine (25 *μ*g/kg), or a combination of methysergide maleate (20 *μ*g/kg) and yohimbine (53 *μ*g/kg). Five minutes after drug injection, the animals were injected with formalin 8% on the hind paw and recording started immediately. The 5 min lapse between drug injection and formalin injection and dose levels were chosen based on published data (Kanui et al. 1993) and preliminary studies. The controls were given saline or DMSO intrathecally.

### Assessment of the sensorimotor performance

Muscle tension and locomotion were used to assess the sensorimotor performance. After the injection of a drug or vehicle, the animal was placed in an observation cage and observed for 1 h. Locomotion of the animal was assessed by monitoring its movement across a marked line drawn on the floor of the cage. To test the muscle tension, a pair of forceps was used to stretch the hind and front feet and the animal's response was noted. Assessment of the effects of the drugs on the tension of the muscle and locomotion was based on arbitrary scale of 0–4 as follows:

0 - No muscle tension/hypoactivity
2 - Normal tension/activity
4 - High muscle tension/hyperactivity

### Data analysis

Data collected following formalin test and antinociceptive testing were tested for equal variance and normal distribution. The data were analyzed using One Way ANOVA with a two sided Dunnets post-hoc test using SPSS 17.0. Sensorimotor performance data were ordinal scale data and the effects of the drugs on sensorimotor performance were analyzed using Kruskal–Wallis ANOVA by ranks. *P*-values lower than or equal to 0.05 were considered statistically significant. For the antinociceptive experiments, results are presented as means ± standard error of the mean (SEM).

## Results

The data demonstrated that formalin (8%), injected intradermally caused a nocifensive behavior that lasted for approximately 20 min. The formalin-induced behavior was effectively reduced by intrathecal administration of clonidine, whose effect was reversed by yohimbine. The antagonistic effect of yohimbine was reversed by methysergide, a 5_HT antagonist.

### Formalin test

**Figure 1:**
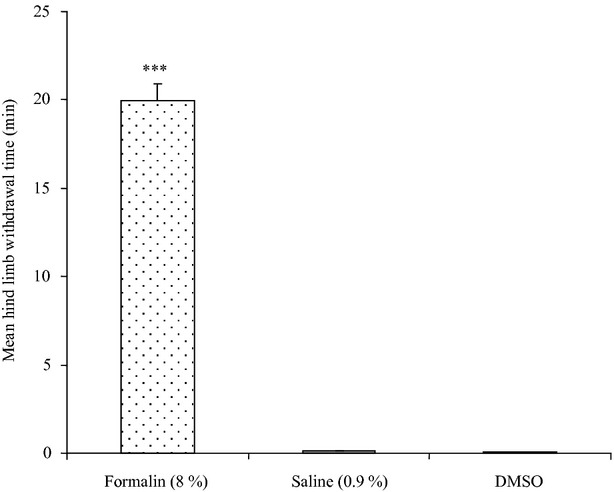
Effects of subcutaneous injection of formalin (8%), saline (0.9%), or Dimethylsulphoxide (DMSO) on the mean hind limb withdrawal time in the marsh terrapin. Bars represent means ± SEM and *n* = 6 in each group. Treatment means were compared using Dunnett's (2-sided) test, subsequent to ANOVA. *** denotes *P* < 0.001 (Saline or DMSO group vs. formalin group).

Subcutaneous injection of 100 *μ*L of formalin (8%) caused pain-related behavior during the first 20 min of the test. The mean time spent in hind limb withdrawal for the formalin group was significantly higher when compared to that for animals injected with saline or DMSO (*P* < 0.001, *F*-test). The mean time spent in pain behavior following formalin injection was 20.32 ± 0.26 min (*n* = 6). Other behaviors observed in a few animals after formalin injection were defecation, salivation, and urination. The mean time spent in pain-related behavior following injection with DMSO or saline was 0.07 ± 0.06 min and 0.12 ± 0.07 min, respectively. Neither DMSO nor saline caused any significant change in the position of the injected limb (Fig.1).

### Effects of Clonidine, yohimbine, and methysergide maleate

**Figure 2:**
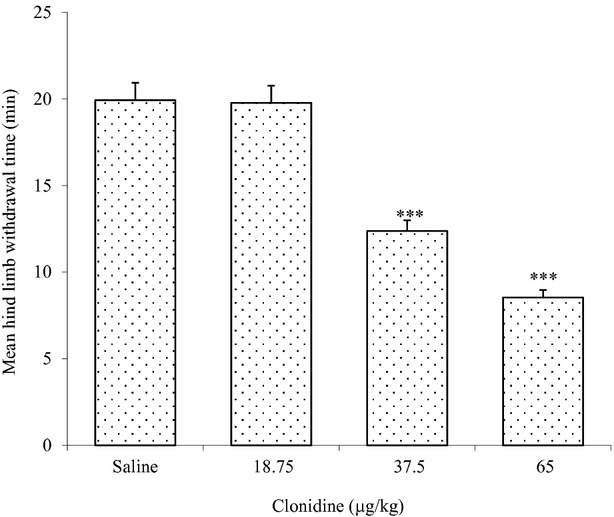
Effects of intrathecal administration of saline or clonidine (18.75, 37.5 or 65 *μ*g/kg) on the mean hind limb withdrawal time in the marsh terrapin. Bars represent means ± SEM and *n* = 6 in each group. Treatment means were compared using Dunnett's (2-sided) test, subsequent to ANOVA. *** denotes *P* < 0.001 (Treatment groups vs. saline group).

The mean time spent in hind limb withdrawal after administration of saline or clonidine at doses of 18.75, 37.5, or 65 *μ*g/kg was 20.45 ± 0.15, 20.16 ± 0.16, 12.38 ± 0.19, and 8.54 ± 0.39 min, respectively. Clonidine at doses of 37.5 *μ*g/kg and 65 *μ*g/kg induced a highly significant [*F*_ANOVA_ (3, 20) = 90.46; *P* < 0.001] decrease in the mean hind limb withdrawal time (Fig.2).

**Figure 3:**
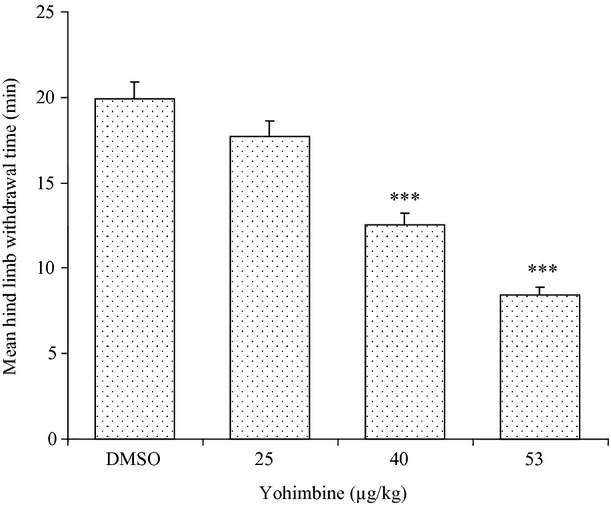
Effect of intrathecal administration of Dimethylsulphoxide (DMSO) or yohimbine (25, 40 or 53 *μ*g/kg) on the mean hind limb withdrawal time in the marsh terrapin. Bars represent means ± SEM and *n* = 6 in each group. Treatment means were compared using Dunnett's (2-sided) test, subsequent to ANOVA. *** denotes *P* < 0.001 (DMSO vs. treated group).

The mean time spent in pain-related behavior after administration of DMSO or yohimbine at doses of 25, 40, or 53 *μ*g/kg was 20.0 ± 0.14, 18.12 ± 0.14, 12.56 ± 0.19, and 8.44 ± 0.19 min, respectively. Yohimbine at doses of 40 *μ*g/kg and 53 *μ*g/kg induced a highly significant [*F*_ANOVA_ (3, 20) = 60.16; *P* < 0.001 decrease in the mean hind limb withdrawal time (Fig3).

**Figure 4:**
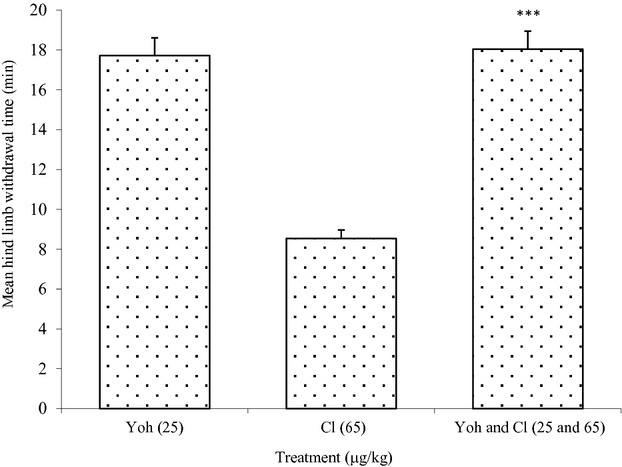
Effect of intrathecal administration of yohimbine (Yoh, 25 *μ*g/kg), clonidine (Cl, 65 *μ*g/kg) or a combination of yohimbine and clonidine (Yoh, 25 *μ*g/kg + Cl, 65 *μ*g/kg) on the mean hind limb withdrawal time in the formalin test in the marsh terrapin. Bars represent mean ± SEM and *n* = 6 in each group. Treatment means were compared using Dunnett's (2-sided) test, subsequent to ANOVA. *** denotes *P* < 0.001 (Clonidine group vs. the combined treatment group).

The mean time spent in pain-related behavior after the administration of yohimbine at a dose of 25 *μ*g/kg followed immediately by that of clonidine at a dose of 65 *μ*g/kg was 18.04 ± 0.23 min. The mean time spent in hind limb withdrawal for the combined treatment (Yoh, 25 *μ*g/kg + Cl, 65 *μ*g/kg) was significantly higher when compared to that of clonidine (65 *μ*g/kg) alone (Fig.4).

**Figure 5:**
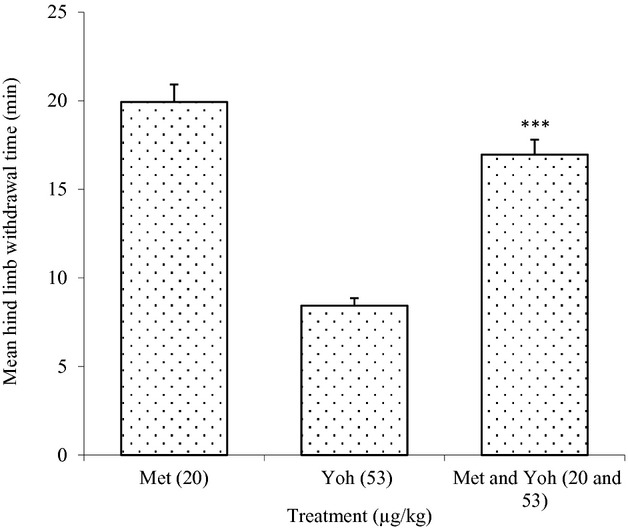
Effect of administration of Methysergide maleate (Met – 20 *μ*g/kg), yohimbine (Yoh, 53 *μ*g/kg), or a combination of methy-sergide maleate (Met – 20 *μ*g/kg) and yohimbine (Yoh, 53 *μ*g/kg) on the mean hind limb withdrawal time in the formalin test. Yohimbine was intrathecally administered immediately after methysergide maleate administration. Bars represent means ± SEM and *n* = 6. Treatment means were compared using Dunnett's (2-sided) test, subsequent to ANOVA. *** denotes *P* < 0.001 (Yohimbine 53 *μ*g/kg group vs. the combined treatment group).

The mean time spent in pain-related behavior after the administration of methysergide maleate at a dose of 20 *μ*g/kg and yohimbine at a dose of 53 *μ*g/kg was 17.35 ± 0.17 min. Injection of the methysergide maleate immediately preceded injection of the yohimbine. The mean time spent in hind limb withdrawal for the combined treatment (Met, 20 *μ*g/kg + Yoh, 53 *μ*g/kg) was significantly higher when compared to that of yohimbine (53 *μ*g/kg) alone (Fig5). Administration of methysergide maleate alone at a dose of 20 *μ*g/kg had no significant effect on hind limb withdrawal time. All the doses of the drugs used in the experiments had no effect on locomotion and muscle tension.

## Discussion

The marsh terrapins showed a nociceptive behavioral response (hind limb withdrawal) following injection with formalin. In the study, 8% formalin was chosen based on preliminary studies. The pain-related behavior lasted for an approximately 20 min. The results indicate that, similar to crocodiles and tortoises (Kanui et al. 1990; Wambugu et al. 2010), marsh terrapins do not show the second phase of pain-related behavior in the formalin test.

In most animals studied, the formalin test is characterized by a biphasic pain response, with distinct first and second phases (Rosland et al. 1990). The first period is commonly referred to as the early phase, and is characterized by short-lasting pain-related responses that last for 5 min post formalin injection. The second phase commences 10–20 min after formalin injection and goes on for the next 30 min. This phase is characterized by continuous prolonged pain-related responses and is usually attributed to inflammatory reactions occurring in the injected area (Dickenson and Sullivan 1987; Shibata et al. 1989; Raboisson et al. 1995). The lack of a second phase in the formalin test in terrapins suggests that the mechanisms regulating this phase may be absent or is different from that of other animals. Perhaps these animals lack neurotransmitters like substance P, Calcitonin Gene-Related Peptide, NMDA, and NK-1 which are known to play a role in the development of the second phase (Allen et al. 2002). The inflammatory reactions necessary for the occurrence of the second phase may be slow or absent in terrapins. Perhaps, in the marsh terrapin the events that occur in the early phase are not adequate to elicit the second phase. The present data do not at the moment provide an adequate explanation as to why terrapins appear not to sense pain after 20 min post formalin inject-ion. This calls for more research in this area of study in chelonians.

In the current study, the experimented drugs were injected intrathecally. This method had not been described before in the marsh terrapins, and therefore this study provided the opportunity to evaluate efficacy of the technique in this species. The method appeared to be effective in drug delivery in this species of reptile. Intrathecal drug injection has been used effectively in other chelonian species (Rivera et al. 2011). In mammalian species, intrathecal injection can be performed in awake, sedated, and anesthetized animals (Stamford 1995). In this study, the intrathecal injection was performed in conscious animals. Although the technique can be used effectively in research setting especially for injection of drugs that do not readily cross the blood brain barrier, it is not advisable for routine use because of its close proximity to the CNS.

The *α*_2_-adrenergic receptor agonist clonidine, administered intrathecally, produced a dose-dependent decrease in the mean hind limb withdrawal time, thereby suggesting antinociceptive effect. This concurs with previous studies in other animal models (Schmitt et al. 1974; Ossipov et al. 1990) and in clinical studies (Mendez et al. 1990; Bernard et al. 1991). For instance, in mice and rats, clonidine has been reported to be as potent as morphine (Fielding et al. 1978), acting both at supraspinal and spinal levels (McCleary and Leander 1981; Yaksh and Reddy 1981; Ossipov et al. 1990). A number of studies have also shown that *α*_2_-agonists, either alone or in combination with local anesthetics or opiate narcotics, are highly effective in the treatment of short-term pain in humans (Kamibayashi and Maze 2000). In addition, alpha agonists have been used successfully for postoperative pain management in surgical populations as diverse as obstetric and pediatric (Tschernko et al. 1998). The use of *α*_2_-agonists in pain management in reptiles and other exotics is advancing especially in reptile surgery (Mader 2006). These data therefore provide additional information. Yohimbine at a dose of 25 *μ*g/kg significantly antagonized the antinociceptive effects of clonid-ine at a dose of (65 *μ*g/kg). The dose (25 *μ*g/kg) of yohimbine used for antagonistic reactions had no significant effect on the mean hind limb withdrawal time when administered alone (Fig.3). This, therefore suggests that the observed antinociceptive effect of clonidine was mediated by an agonistic action at *α*_2_-adrenergic receptors.

Clonidine inhibits nociceptive transmission by mimicking the action of spinally released norepinephrine from descending noradrenergic inhibitory pathways (Stamford 1995). The inhibition of nociceptive transmission in the dorsal horn seems to be mediated particularly via presynaptic *α*_2A_ receptors in primary nociceptive terminals (Samuels and Szabadi 2008). The existence of noradrenergic system has been documented in many vertebrates including quadrupedal reptiles such as the turtles *Pseudemys scripta elegans* and *Testudo hermanni* and the lizards *Tupinambis nigro punctatus* and *Varanus exanthematicus* (Donkelaar et al. 2004). The origin and distribution of the noradrenergic neurons in these reptiles are similar to those of mammals (Donkelaar et al. 2004). On the basis of these reports, it is most likely that the marsh terrapins are no exception.

Several studies have indicated that yohimbine does not completely antagonize the effects of clonidine in the formalin test (Tasker and Melzack 1989; Shannon and Lutz 2000), but instead it may act as an agonist (Shannon and Lutz 2000). This agrees well with the current study where the higher doses (40 or 53 *μ*g/kg) of yohimbine had antinociceptive effects. This is also in agreement with reports where i.t. yohimbine inhibited nociception in the hot plate and the formalin tests in rats and mice (Dennis et al. 1980; Kanui et al. 1993). The agonistic effect of yohimbine is suggested to be mediated by a partial agonistic effect on 5-HT_1A_ receptors (Shannon and Lutz 2000) as supported by this study. In the study the nonselective 5-HT receptor antagonist, methysergide maleate, administered at a dose of 20 *μ*g/kg significantly antagonized the antinociceptive effect of yohimbine (53 *μ*g/kg). This suggests the existence of 5HT receptors in the marsh terrapin. This calls for a systematic study to evaluate the significance of serotonergic system in pain regulation in testudines.

Although *α*_2_ agonists including clonidine have proved to be effective analgesics, they do also have a number of side effects that seem largely to depend on the dose volume. Clonidine, for instance induces hypnosis (Mizobe et al. 1996), and altered thermoregulation (LoPachin and Rudy 1981). The reported side effects were not measured in this study and might not have been there because the treated animals were behaviorally similar to controls. Clonidine may also cause motor blockade in experimental animals (Klimscha et al. 1995). However, in the marsh terrapins, the drugs used had no effect on sensorimotor performance which was measured as described in the materials and methods section. This concurs with previous findings that smaller doses of clonidine and yohimbine had no effect on the sensorimotor performance in the crocodiles (Kanui et al. 1993). However, it has been reported that systemically administered antinociceptive doses of clonidine significantly impaired motor coordination in mice (Dogrul and Uzbay 2004). This could be due to the species difference.

In conclusion, the study shows that the formalin test is a good test for studying nociception and anti-nociception in the marsh terrapin, but more data are required to address the mechanisms behind the lack of the chronic pain in this animal. The study also showed that intrathecal method of drug delivery is applicable and effective for drug administration in the marsh terrapin. The current data clearly show that clonidine and yohimbine administered intrathecally appear to be good analgesic drugs for use in the terrapins. The study further indicates that the noradrenergic and serotonergic systems appear crucial in pain regulation in the marsh terrapins. These animals appear to be a good model for investigating analgesic effects of drugs that can be used for both animals and humans.
